# Trade protectionism and the manufacturing sector: a review of border closure policy in Nigeria

**DOI:** 10.1186/s43093-022-00170-4

**Published:** 2022-12-12

**Authors:** Olufunke Iyabo Ajoje, Olufemi Samuel Adegboyo

**Affiliations:** Department of Economics, Federal University Oye-Ekiti, Oye-Ekiti, Ekiti Nigeria

**Keywords:** Trade protectionism, Free trade, Border protection, Manufacturing output, F13, F14, F19, O14

## Abstract

This study empirically investigates to ascertain the impact of trade protection vis-à-vis border closure policy on manufacturing sector in Nigeria between January 2018 and June 2021 using monthly secondary data. The study employs traditional theory of protectionism as its theoretical framework. The chow breakpoint result revealed that there is a significant change in the parameters of the model in July 2019 which coincides with the time the policy implementation started. It employs dummy variable to investigate the impact of the policy on manufacturing sector output as against use of two regression model. The regression analysis revealed that in the short run, the impact of the border closure on manufacturing sector was positive but later became adverse in the long run. Also, the interaction of the border closure with the inflation rate revealed that the inflation rate became high during the period but the government generated income from tariff increased. This revealed that there are leakages through the land borders that needs to be curbed through legislation. Sequel to these findings, the study makes the following recommendations: government should not consider closing the borders again as it closures constitute a drag to the manufacturing sector growth; rather than closed border, government should formulate policies to enforce trade protection; lastly, should it become exigent for the government to close the border, they should allow moderate inflation rate that the economy can tolerate in order to spur manufacturing output.

## Introduction

The under performance of the Nigerian manufacturing sector vis-à-vis its contribution to gross domestic product (GDP) has often been attributed to influx of foreign products that can be produced locally which occurs mostly through the land borders. In an attempt to revive the manufacturing sector, the government in the third quarter of 2019 closed all its land borders restricting all manufactured goods especially rice, poultry products and textiles that can be produced locally from being imported in to the country especially through the land border. The justification by the government was premised on the ground that it has been difficult for the agricultural and manufacturing sector to function optimally due to influx of foreign products that can be produced locally; that the land borders has been a medium of evading duties especially at the Benin Republic axis of the country which has majorly become an entrepôt for already manufactured goods which have the final destination as Nigeria [1]. Goods are also routed through the Benin axis in order to evade import duties and quality assurance. The government also maintained that the land borders have been a channel through which illegal arms get into the country and this therefore made the combating insecurity an uphill task coupled with being the media for illegal exporting of subsidised petroleum products which has a devastating effect on the economy [2].

Despite the justification of this policy, critics have maintained that Nigeria is both a sovereign, developing country and also a member of many international organisations such as Economic Community of West African States (ECOWAS), African Union (AU) and World Trade Organisation (WTO) which support free trade and that she also signed the free trade agreement as a member of the African Continental Free Trade Area (AFTFCA). In light of this, the policy was viewed by the critics as a violation of agreements of these international organisations and most especially just signed AFTFCA as the tenets of these organisations contradict the operation of Trade protectionism [3]. The critics also maintained that the world economy has become increasingly linked through expanded international trade in services, primary and manufactured goods, international portfolio investments thereby encouraging importing and exporting of these goods among nations and that closing these borders would only mean dragging the Nigerian economy back to the stone age Ugwuja and Chukwukere [4]. Despite these arguments, the federal government has maintained that no criticism can sufficiently supersede the issue of insecurity, unemployment and protection of local manufacturing industries which dominates the manufacturing sector and the economy as a whole.

The viability of the manufacturing sector of any economy plays a major role in the development of the country and its role in National output growth cannot be over-emphasized. In developed countries such as the United State of America, United Kingdom, Malaysia and China manufacturing sector contributes a substantial percentage of the GDP at times as high as between 17 and 20% [5]. This is not unconnected with the rate of growth and development in these countries.

In the case of Nigeria, the manufacturing sector has been saddled with issues such as smuggling, dumping and international price competition which has almost strangled the few viable manufacturing companies in the country. In order to salvage this, different industrialisation strategies such as import substitution, export promotion among others have been employed. Despite all this, the manufacturing sector has contributed within the range of 4.5–12.67% of the GDP in the last 10 years [5]. This is not unconnected with the illegal means of importation especially through the land borders as well as poor trade and development policies in the country [6].

Having realised the effect of these vices on the economy by the federal government, the Central Bank of Nigeria was in 2016 mandated to carry out a survey on informal cross-border trade (ICBT) to ascertain the extent of economic loss owing to these vices as a que from other countries which have successfully measured their ICBT. Such countries include; Malawi, Uganda and Mozambique. The report revealed that ICBT though has not been properly documented, it remains a major source of income, employment and food security for a non-negligible percentage of the country's population as well as a major income leakage source to the government. It was also revealed that the volume and magnitude of the ICBT between Nigeria and her neighbouring countries which occurred as a result of illegality though not included in the Balance of Trade Statistics has great impact on the economic decisions of the government [7].

The Central Bank of Nigeria in his report therefore maintained that the decision of the government to close the land borders was long overdue as the impact of smuggling activities especially in rice and petroleum products has taken its negative toll on the economy going by the outcome of the survey [8]. It was also established by the Nigeria Customs service that the income generation in the area of duties and tariffs from illegal trade is as much as 500% of the legitimate importation. This border closure therefore resulted in income generation increase for instance from 4.5 billion Naira in July 2019 to as high as 115 billion naira in September 2020 and the trend has continued [9].

This study will therefore access the performance of the manufacturing sector before during and after the closure in order to appraise the impact of the policy on the manufacturing sector as a whole. The study will cover the period before, during and after the policy in order to appraise the policy impact.

## Literature review

This section of the study is divided into three sub-sections, namely Conceptual, theoretical and empirical review.

### Conceptual review

#### Trade protectionism

The concept of trade protectionism otherwise known as trade barrier is as old as international trade itself and it has been defined by different authors from different perspectives but the major point of consensus in the various definitions is defining trade protectionism as all government policies both in theory and practice which restricts imports from other countries through the application of different methods such as import restrictions, quotas among others with the major aim of protecting local industries by limiting foreign goods sold in the market place. It is usually implemented to enhance the economic activities within the domestic economy by increasing local production and also shielding the local economy from international trade competition which in most cases is not able to successfully compete with especially in the developing countries.

### Manufacturing output

Manufacturing can be defined as the physical process that enhances economic value of raw materials and converts to finished goods [10]. The output from such process is referred to as the manufacturing output. The concept of manufacturing output therefore covers all the production of final or intermediate goods generated from factories across the country which in most cases are meant for satisfaction of human wants [11].

## Theoretical review

Trading among nations is as old as the World itself and it has witnessed an increased pace after the Second World War. The establishment of General Agreement on Tariffs and Trade (GATT) and the WTO was premised on the fact that free trade between and among nations of the World would enhance stability both politically and economically [12] and that it encourages specialisation. Other than this, the major issue under consideration of free trade has been protection of local industries and maintenance of national sovereignty which has a very high tendency of being compromised. Thus, countries who engage in free trade need to guide it with international regulations in order not to harm their local industries as the country is said to have trade advantage when the economic welfare is enhanced [13]. Such regulations come in form of tariffs, quota, import licenses, import ban and foreign exchange control. This has therefore come under serious arguments and scrutiny by different theories in support and against protectionism. There have been various arguments in support and against protectionism as a tool of protecting local industries. Therefore, this paper will review some of the trade theories that centres on protectionism and economic integration.

## Theories of protectionism

### Traditional theory of protectionism

Economist and welfare analysts have always been in support of free international trade as no country can operate in isolation but the popularity of protectionism in the early 1980s was also borne out of various arguments one of which is the infant industry argument which acknowledges that developing countries have a potential comparative advantage in manufacturing but that these manufacturing companies cannot effectively compete with the organised and well developed manufacturing sector in the developed countries [4]. The major assumptions include the assumption of full employment, fixed production technology, balanced trade, perfect mobility of factors within and among nations and that, international prices are controlled by forces of demand and supply [14]

The justification for theory of protectionism was analysed by [15] and it was centred on the traditional approach to protectionism. This theory centres on the tendency of the government to seek for maximisation of income through protectionism. The basic tenet of the theory is that government seeks to achieve certain non-economic objective through the application of protectionism and also seeks to maximise real income. This was criticised in 1968 [16] on the ground that Johnson failed to consider the welfare aspect of international trade in his analysis and its inability to measure traditional welfare loss as a measure of divergence of social from private costs or benefits of protectionism.

This theory therefore opined that protectionism is a mean of supporting new industries in order to enable them grow strong in order to effectively meet international competition. Even though there have been compelling arguments in favour of free trade and increased trade openness especially after the second World War, the World Trade pattern has to a large extent, been shaped by protectionism in recent years.

In the case of Nigeria, this study opined that although the government considered income leakage through illegal importation and smuggling and protection of local industries, analysts have opined that the welfare aspect of the policy was not considered owing to the fact that local production cannot be sufficiently enough to meet local needs [12].

In most developing countries including Nigeria, International Trade policies are mostly guided by the principle that a strong and vibrant manufacturing sector is a major key to Economic Development and that this can best be achieved by protecting the local manufacturers from international competition which is theoretically and practically a tool to enhance local production Bello et al. [17]. The theory has been criticised based on its basic assumptions and a new theory was propounded by David Ricardo.

### Ricardian theory of protectionism

Contrary to the traditional theory, Coughing et al. [18] premised his argument on David Ricardo’s theory of 1817 in “Principle of political economy and Taxation” and employed this to explain the benefits of free trade. He therefore opined that the concept of protectionism which only became popular in the 1980s does not encourage specialization Ffrench-Davis et al. [19]. His argument was based on perfectly competitive market and that protectionism can lead to price increase in such markets. He therefore concluded that even though import reduction increases local employment especially in industry that provide similar products, export reduction also reduces employment in industries that specialise on exportation of their products and that developing countries also need exports to pay off their debts. This therefore indicates that while protectionism is beneficial to import based industries, it is detrimental to export based industries. This further corroborated the opinion of [20] that jobs saved by protectionism are more than the job lost.

### Theoretical review on manufacturing output

The two major theory that relates to manufacturing output is the Kaldor first and second growth theory. The theories relate manufacturing output, economic growth and external sector of the economy.

#### Kaldor’s first theory

This was postulated in 1949 with the basic tenets that manufacturing output and growth of any economy are positively and closely related.

He postulated that expansion in the manufacturing sector will lead to employment which will further lead to transfer of labour from the low productivity sector to the industrial sector. He further stated that productivity growth rate is endogenous and depends on output growth rate capturing dynamic contexts, endogeneity of the factors and increasing industrial economies of scale. This implies that the development of the local economy will impact positively on the manufacturing sector. That is, the development of the local economy will improve the availability of raw materials which will in turn reduce over-dependence on foreign markets.

#### Kaldor’s second theory

This is also known as the Verdoon’s law and was postulated in 1966. This law established the statistical relationship between the growth of manufacturing output and labour productivity growth on manufacturing where causality runs from former to the latter. Its basic argument is that an initial growth in output labour cost given a markup pricing rule for a fall in prices increasing the competitiveness of a country. These gains in turn allow for further output expansion through increasing exports which reinitiate the cycle. This implies that once a country or region acquires a growth advantage, it will tend to keep it through the process of increasing returns and consequent competitive gains that growth itself induces.

### Emerging theory of manufacturing

Drucker [21] criticised the Kaldor’s law and opined that manufacturing output impact is not only on economic growth but that it is the integrator that connects all sectors of the economy. He further corroborated this that manufacturing sector provides economic value that pays for everything and everybody. Even though he agreed with the Kaldor’s law on its impact on economic growth, he went further to affirm that its greatest impact covers both social and human concerns.

### Empirical literature

Li and Whilley [22] examined the relationship between Trade protectionism and manufacturing sector employment in the United States of America (USA) over the period of 1976–2008 employing the General equilibrium model. The study established that trade protectionism could increase the demand for USA domestic manufactured goods as a result of decreased foreign demand but the simulation results showed that USA trade protectionism reduced manufacturing sector employment.

Ugwuja and Chukwukere [4] examined the concept of trade protectionism and border closure in Nigeria from the political economy perspective by reviewing the rice production from 1984 till date. This study established that even though trade protectionism will boost domestic economy which is in line with the international trade theory, not all stakeholders will benefit from the as there was evidence of food inflation within the period under review.

Cheng et al. [23] studied the impacts of trade protectionism on the Indian economy especially on the manufacturing sector from 1970 to 2017 using the ordinary least square (OLS) econometric technique. The study opined that there are two sides to the trade protectionism theory. In their analysis, it was stated that even though trade protectionism provides a less competitive market for domestic industries and provides a relatively stable environment for their growth; encourage exports and also increase revenue to the government, the policy may not be sustainable as the high tariff rate will eventually harm the economy GDP.

Barratieri et al. [24] examined the macroeconomic impact of trade protectionism using the estimates from country level as well as setting up a small open economy model with firm heterogeneity and employed the Vector autoregressive technique. The study revealed that the policy is not an effective tool in stimulating a macroeconomic environment of a country especially the developing ones.

Abegunde and Fabiyi [25] reviewed the implication of the recent Nigeria-Benin border closure on Nigeria economic development. He employed the OLS technique and affirmed that border closure which had the major aim of protecting the economy has only increased smuggling which are carried out by citizens of both countries. He also established that domestic production, income and patronage increased with reduced national fuel consumption and increased seizure of contraband goods. He therefore concluded that the border closure was not to the economic development of Nigeria.

Eselebor [1] reviewed the effect of Benin-Nigeria Border closure which has been closed in four times between 1984 to March 2020 on safety and vulnerability of the two countries. He therefore concluded that the closure of the border which is the busiest commercial gate way in West Africa has really not curbed smuggling and other security concerns but has opened more bush pathways and maritime routes for the menace. He therefore concluded that the securitisation and better management of the border would be more beneficial to both countries other than the border closure.

Aniukwu [26] examined the impacts and prospects of border closure policy in Nigeria especially in rice production and compared with other nations such as China, Singapore and Japan that have implemented such policy in the past. He therefore concluded that the rice sellers practiced monopoly thereby causing price increase. Although the farmers and the rice sellers enjoyed increased income, it impacted negatively on the cost of living of the citizens.

Kituyi [27] reviewed the performance of the countries in the United Nation which adopted the trade protectionism especially during the COVID-19 period. He analysed that even though trade protectionism may be a tool for building a resilient economy, the growth may not be sustainable as protectionism will only provide temporary relief. It often leads to price hike and supply shortages in the international market. He opined that any economy trying to recover should only adopt it for a while as the manufacturing sector output will not sustain the economy.

Okere and Iheanacho [28] studied the indirect impact of trade protectionism on Nigeria’s economic growth from 1990 to 2013. The Autoregressive distributed lag Bound testing approach was applied to cointegration. In the analysis, protectionism was measured with three variables which are: Trade openness, Subsidy and real exchange rate while the indirect impact on economic growth was measured through industrial production and level of unemployment. The results confirmed the existence of long run relationship but there was no evidence of long causal relationship between real GDP, industrial production, unemployment and labour but in the short run, there is a unidirectional causal relationship running from GDP per capita from industrial production to labour. This study contradicts the general opinion that protectionism is detrimental to economic growth as proved empirically by revealing an indirect link between protectionism and economic growth through industrial production and economic growth.

David et al. [29] reviewed the interrelationship between trade barriers and economic growth with the main objective to determine the nature of the relationship that exist between the duo over the period of 1970–2006. He employed the OLS regression techniques. This revealed that trade openness and economic growth are positively related such that tariff barrier will positively affect economic growth and export while negatively impacts imports.

### Identified research gap

Recent studies in the area of protectionism have linked the concept to increased domestic production coupled with increase in prices of goods and services. This is in line with the traditional protectionism theory which postulated that international competition limits the efficiency of the domestic economy. The reviewed literatures therefore revealed that even though trade protectionism might be beneficial in the area of income generation to the government, it is not all beneficial to the citizens and that in most cases do more harm than good in the area of smuggling and high cost of living.

Previous studies have focused on particular subsector of manufacturing industry. Even though this study is in line with the previous studies, it goes further to review extensively the impact of the policy on the manufacturing sector which the government tried to protect and also employed different econometric approach from the previous studies.

## Methodology

### Theoretical framework

This study adopts the traditional theory of protectionism which supports free international trade as the proponents opined that no country can operate in isolation but the popularity of protectionism in the early 1980s was also borne out of various arguments one of which is the infant industry argument which acknowledges that developing countries have a potential comparative advantage in manufacturing but that these manufacturing companies cannot effectively compete with the organised and well developed manufacturing sector in the developed countries Ugwuja and Chukwukere [4].

The justification for theory of protectionism was analysed by [15] and it was centred on the traditional approach to protectionism. This theory centres on the tendency of the government to seek for maximisation of income through protectionism. The basic tenet of the theory is that government seeks to achieve certain non-economic objective through the application of protectionism and also seeks to maximise real income. This was criticised in 1968 [16] on the ground that Johnson failed to consider the welfare aspect of international trade in his analysis and its inability to measure traditional welfare loss as a measure of divergence of social from private costs or benefits of protectionism [30].

This theory therefore opined that protectionism is a mean of supporting new industries in order to enable them grow strong in order to effectively meet international competition. Even though there have been compelling arguments in favour of free trade and increased trade openness especially after the second World War, the World Trade pattern has to a large extent, been shaped by protectionism in recent years [31].

In the case of Nigeria, this study opined that although the government considered income leakage through illegal importation and smuggling and protection of local industries, analysts have opined that the welfare aspect of the policy was not considered owing to the fact that local production cannot be sufficiently enough to meet local needs [12].

In most developing countries including Nigeria, International Trade policies are mostly guided by the principle that a strong and vibrant manufacturing sector is a major key to Economic Development and that this can best be achieved by protecting the local manufacturers from international competition which is theoretically and practically a tool to enhance local production [17]. This therefore indicates that while protectionism is beneficial to import based industries, it is detrimental to export based industries.

This further corroborated the opinion of [20] that jobs saved by protectionism are more than the job lost.

### The model

Based on the theoretical framework, the model is specified below:

1 $$Y = f\left( {{\text{TRPT}}} \right)$$

where *Y* = output, TRPT = trade protection.

This research work will add some control variables which extant studies such as…have identified as factors that influence manufacturing output. Consequently, the model is specified below:

2 $${\text{MANGR}}\,\, = \,\,f\left( {{\text{TPRT}},\,\,{\text{GNPGR}},\,\,{\text{INFR}}} \right)$$

The econometric specification is therefore.

3 $${\text{MANGR}}_{t} = \beta_{0} + \beta_{1} {\text{TPRT}}_{t} + \beta_{2} {\text{GNPGR}}_{t} + \beta_{3} {\text{INFR}}_{t} + \mu_{t}$$

where MANGR is the manufacturing sector output growth rate. TPRT is the trade protection (proxied income generated by Customs from government levied tariffs). GNPGR is the gross national product growth rate. INFR is the inflation rate. *µ* is the error term. *t* is the time trend. *β*_0_–*β*_3_ are the vector of the parameters/variables.

There is need to transform Eq. 3 for all the variables to be in the same appropriate coefficient as variables such as manufacturing sector output growth rate, gross national product growth rate and inflation rate were in rate while trade protection was in billions as such the model will be transformed to a log-linear model which is presented in Eq. 4.

4 $${\text{MANGR}}_{t} = \beta_{0} + L\beta_{1} {\text{TPRT}}_{t} + \beta_{2} {\text{GNPGR}}_{t} + \beta_{3} {\text{INFR}}_{t} + \mu_{t}$$

### Apriori expectation and justification

Based on the adopted theory, protectionism is expected to have a positive relationship with manufacturing sector output production due to reduced importation and increased production, as well as the National Output and generated government revenue.

Gross national product growth rate is expected to have a positive impact on manufacturing output because as the national income increases, the purchasing power will increase and this will translate to higher demand which will stimulate manufacturers to increase their production.

Inflation rate is expected to have a negative impact on manufacturing output because it will increase the cost of raw materials and consequently increase production cost which will in turn reduce demand for the goods.

## Result and discussion

### Descriptive analysis

This section begins with the descriptive analysis of the variables under study. This is done in order to explore the characteristics of each variables the study employs. The summary of the descriptive analysis is presented in Table 1. The table shows that the mean (average value) of manufacturing sector output growth rate, trade protection, inflation rate and gross national product growth rate are 10.138, 10.459, 13.017 and 0.161, respectively. The table also shows that the mean value of manufacturing sector output growth rate, trade protection and gross national product growth rate were smaller than their median value that is the values manufacturing sector output growth rate, trade protection and gross national product growth rate are higher than their average value which implies that the distribution are skewed to the left. Furthermore, the mean value of inflation rate is greater than its median indicating that inflation rate is skewed to the right. Also, the table shows the standard deviation which is used to measure the stability or otherwise of the variable reveals that GNPGR which has the highest value is the most unstable variable while trade protection which has the lowest is the most stable variable. The table also shows that all the variables displayed a high degree of consistency because their median and mean value fall within the maximum and minimum values of the variables. Furthermore, skewness statistic shows that manufacturing sector output growth rate, trade protection and gross national product growth rate were negatively skewed towards normality while inflation rate was positively skewed towards normality. In addition, the kurtosis that measures the peakness or otherwise of the distribution reveals that all the variables were platykurtic except inflation rate, this implies that all the variables were flat in relative to normal distribution except inflation rate. Finally, the Jargue–Bera statistics shows that all the variables were normally distributed at 5% significant level aside inflation rate.

**Table 1:** Summary of descriptive analysis

	MANGR	LOG(TPRT)	INFR	GNPGR
Mean	10.138	10.459	13.017	0.161
Median	10.355	11.655	12.165	3.02
Maximum	15.27	12.094	18.17	12.12
Minimum	4.36	8.446	11.02	− 14.27
Std. Dev	3.191	1.532	2.255	9.043
Skewness	− 0.357	− 0.125	1.201	− 0.608
Kurtosis	1.863	1.084	3.111	1.902
Jargue–Bera	3.152	6.534	10.109	4.695
Probability	0.207	0.381	0.006	0.096

Source: Authors’ computation

### Correlation analysis

This is used to analysis the relationship between variables. The result of the correlation is presented in Table 2. The result reveals that manufacturing sector output growth rate has a strong positive relationship with trade openness. The result also shows that manufacturing sector output growth rate has a weak positive relationship with inflation rate but had a weak negative relationship with gross national product growth rate. Furthermore, the result reveals that trade protection had a weak and positive relationship with inflation whereas it has a weak and negative relationship with gross national product growth rate. Lastly, inflation rate was found to have a weak negative relationship with gross national product growth rate.

**Table 2:** Result of correlation matrix

	MANGR	LOG(TPRT)	INFR	GNPGR
MANGR	1			
LOG(TPRT)	0.885	1		
INFR	0.499	0.496	1	
GNPGR	− 0.318	− 0.375	− 0.308	1

Source: Authors’ computation

### Unit root test

The study conducts the stationarity test to determine the level of stationarity of each variable. The study employs Augmented Dickey–Fuller test and Phillips–Perron test to determine the stationarity of each variable.

The result of the stationarity tests is presented in Table 3. The two test presents similar result. The two results reveal that all the variables are not stationary at level aside gross national product growth rate which became stationary only at 10% significant level. The results also show that all the variables became stationary at the first difference.

**Table 3:** Result of unit root test

Variables	ADF	*P*–*P*
*At level*		
GNPGR	− 2.843*	− 2.843*
INFR	− 1.884	− 0.289
MANGR	− 1.959	− 2.353
TPRT	− 1.096	− 1.096
*First difference*		
GNPGR	− 5.714***	− 6.171***
INFR	− 4.206***	− 4.271***
MANGR	− 8.069***	− 16.454***
TPRT	− 5.122***	− 5.061***

Source: Authors’ computation

Significant level: * = 10%, ** = 5%, *** = 1%

### Chow test

Sequel to the objective of the study which is to examine the effect of trade protection policy in 2019 vis-à-vis broader closure, therefore this study will employ chow test as it is the estimate that allow for breakpoint/structural change in the regression analysis. Having identified the appropriate technique to be adopted, it is sacrosanct to determine if truly there is a break in the series. Since the policy was introduced in July 2019 therefore it will be used as the breakpoint. The result of the chow breakpoint test is presented in Table 4. The table reveal that the F-statistic is significant which implies that the null hypothesis of no break at July 2019 is rejected. This indicates that there is a significant change in the parameters of the model in July 2019. This denotes that there is a significant difference between the impact of trade protection on manufacturing output before the border closure and after the closure.

**Table 4:** Chow breakpoint test

*F*-statistic	2.533	Prob *F*	0.0581
Log likelihood ratio	10.956	Prob. chi-square	0.0271
Wald statistic	10.134	Prob. chi-square	0.0382

Having identified that there is breakpoint in the model, this study will employ dummy variable to investigate the impact of the policy on manufacturing sector output as against using of two regression model. This technique solves the problem of degree of freedom that the usage of two analysis will encounter. For the dummy variable, pre-broader closure will be represented by 0, while post-broader closure will be captured by 1.

5 $${\text{MANGR}}_{t} =\, \beta_{0} + L\beta_{1} {\text{TPRT}}_{t} + \beta_{2} D_{t} + \beta_{3} {\text{GNPGR}}_{t} + \beta_{4} {\text{INFR}}_{t} + \mu_{t}$$

In accessing how the policy affects manufacturing sector output growth rate this study will interact the dummy variable with all the independent variables and it is specified in Eq. 6

6 $$\begin{aligned} {\text{MANGR}}_{t} = &\, \beta_{0} + L\beta_{1} {\text{TPRT}}_{t} + \beta_{2} D_{t} + \beta_{3} {\text{GNPGR}}_{t} + \beta_{4} {\text{INFR}}_{t} \\ & + L\beta_{5} D_{t} {\text{TPRT}}_{t} + \beta_{6} D_{t} {\text{GNPGR}}_{t} + \beta_{7} D_{t} {\text{INFR}}_{t} + \mu_{t} \\ \end{aligned}$$

The result in Table 5 reveals that trade protection has a positive and significant impact on manufacturing output, this implies that trade protection spur manufacturing output in Nigeria. It further reveals that 1% increase in trade protection will lead to 0.9733% increase in manufacturing output in Nigeria. This conforms with the apriori expectation. This could be because the protection prevents the country from becoming a dumping ground or because it prevents importation of goods that can be produced locally.

**Table 5:** Result of the regression analysis

Variable	Coefficient	Std error	*t*-statistic	Prob
Log(TPRT)	0.9733	0.1079	2.5605	0.0191
INFR	− 0.5462	0.5349	− 5.8736	0.0001
GNPGR	0.0033	0.9849	4.5754	0.0002
Dummy	− 3.2872	0.0423	− 3.6726	0.0016
Log(TPRT) × dummy	− 0.4032	0.0198	− 3.5992	0.0019
INFR × dummy	0.8363	0.0351	3.3055	0.0037
GNPGR × dummy	− 0.0044	0.1956	− 0.2589	0.7984
C	5.0479	0.0237	2.2399	0.0372
*R*-square	0.8317			
Adjusted *R*-square	0.8002			
Durbin–Watson	1.9651			

Furthermore, the result in the table shows that inflation rate a negative and significant impact on manufacturing output, this implies that inflation rate deter manufacturing output in Nigeria. The result further shows that 1% increase in inflation rate will leads to 0.5462% decrease in manufacturing output in Nigeria. This conforms to the apriori expectation. This could be because inflation rate will lead to high cost of doing business which will in turn reduce business turnover.

The result also reveals that gross national product growth rate has a positive and significant impact on manufacturing output. This denotes that as the economy grows, the manufacturing sector output also increases. The result also shows that 1% increase in gross national product growth rate will lead to 0.0033% increase in manufacturing output in Nigeria. This conforms with the apriori expectation.

Contrariwise, the border closure policy which the dummy variable represents shows that border closure had a negative and significant impact on the manufacturing output in Nigeria this implies that manufacturing output deteriorate after the border closure that it was prior to the closure. The result also shows that 1% increase in the border leads to 3.2872% decline in manufacturing output. This could be because the manufacturing sector relies on imported raw materials to process their goods.

Similarly, the result reveals that the interaction of the dummy variable (border closure) and trade protection had a negative and significant impact on manufacturing output in Nigeria, this implies that border closure vis-à-vis trade protection constitute a drag to the manufacturing output in Nigeria. The result also denotes that, border closure policy derails the benefit that manufacturing sector are benefiting from trade protection. The result also reveals that 1% increase in the interaction of dummy variable (border closure) and trade protection leads to 0.4032% decrease in manufacturing output in Nigeria, this indicates that border closure drags the benefits that manufacturing sector gets form trade protection before the closure by 0.4032%.

The result reveals that the interaction of the dummy variable (border closure) and inflation rate had a positive and significant impact on manufacturing output in Nigeria, this implies that border closure vis-à-vis inflation rate stimulates manufacturing output in Nigeria. This indicates that border closure neutralized the adverse effects of inflation rate on manufacturing output in Nigeria. The result also reveals that 1% increase in the interaction of the dummy variable (border closure) and inflation rate leads to 0.8363% increase in manufacturing output, this denotes that border closure enhance inflation rate to promote manufacturing output by 0.8363% compare to pre-border closure.

The interaction of the dummy variable (border closure) and gross nation product growth rate was found to have a negative but not significant impact on manufacturing output in Nigeria.

The model displayed a good fit at its adjusted *R*^2^ value is 0.8002 which implies that about 80.02% of the variation in manufacturing output are been explained by explanatory variables.

Lastly, the result of the Durbin–Watson test reveals that there is no problem of autocorrelation.

## Conclusions

The under performance of the manufacturing sector vis-à-vis its contribution to gross domestic product has often been attributed to influx of foreign products that can be produced locally and this often happen through land borders. Also, the land borders have been a medium of evading duties especially at the Benin Republic axis of the country which has majorly become an entreport for already manufactured goods. As such the country is deprived in two sides: country becoming dumping ground, and losing traffic to smugglers. In an attempt to revive the manufacturing sector, the government in the third quarter of 2019 closed all its land borders. This study then empirically investigates to ascertain the impact of trade protection vis-à-vis border closure on manufacturing sector in Nigeria between January 2018 and June 2021 using monthly data. The study employs traditional theory of protectionism as its theoretical framework. The study used income generated by Customs from government levied tariffs as proxy for trade protection. The work adopts two different unit root tests, namely Augmented Dickey–Fuller and Phillips–Perron tests. The two test presents similar result. The two results reveal that gross national product growth rate is stationary at level at 10% significant level. The results also show that inflation rate, manufacturing sector output growth rate, and trade protection were not stationary at level but they all became stationary at the first difference. The chow breakpoint result reveals that there is a significant change in the parameters of the model in July 2019 which implies that there is a significant difference between the impact of trade protection on manufacturing output before the border closure and after the closure. This employs dummy variable to investigate the impact of the policy on manufacturing sector output as against using of two regression model. This technique solves the problem of degree of freedom that the usage of two analysis will encounter. For the dummy variable, pre-broader closure was represented by 0, while post-broader closure was captured by 1. The regression analysis reveals that border closure and the interaction of the border closure and trade protection adversely affects manufacturing sector, the interaction of the border closure and inflation rate stimulates manufacturing output while the interaction of the border closure and gross nation product growth rate had no impact on manufacturing output. Sequel to the findings, the study makes the following recommendation: government should not consider closing the borders again as it closures constitute a drag to the manufacturing sector growth as against the intension of stimulating manufacturing sector; rather than closed border, government should formulate policies to enforce trade protection; lastly, should it become exigent for the government to close the border, they should allow moderate inflation rate that the economy can tolerate in order to spur manufacturing output.

## Data Availability

This is available on request.

## Abbreviations

ECOWAS: Economic Community of West African States
AU: African Union
WTO: World Trade Organisation
AFTFCA: African Continental Free Trade Area
GDP: Gross domestic product
ICBT: Informal cross-border trade
GATT: General Agreement on Tariffs and Trade
USA: United States of America
OLS: Ordinary least square
MANGR: Manufacturing sector output growth rate
TPRT: Trade protection
GNPGR: Gross national product growth rate
INFR: Inflation rate
